# Does the gap between health workers’ expectations and the realities of implementing a performance-based financing project in Mali create frustration?

**DOI:** 10.1186/s41256-021-00189-0

**Published:** 2021-02-02

**Authors:** Tony Zitti, Amandine Fillol, Julia Lohmann, Abdourahmane Coulibaly, Valéry Ridde

**Affiliations:** 1 Centre Population et Développement (Ceped), Institut de recherche pour le développement (IRD) et Université de Paris, Inserm ERL 1244, 45 rue des Saints-Pères, 75006 Paris, France; 2 École doctorale Pierre Louis de santé publique, Université de Paris, Paris, France; 3 ONG Miseli, Bamako, Mali; 4 Ecole de santé publique de l’Université de Montréal, Montréal, Canada; 5 London School of Hygiene & Tropical Medicine, London, UK; 6 Heidelberg Institute of Global Health, Medical Faculty and University Hospital, Heidelberg University, Heidelberg, Germany; 7 Faculté de Médicine et d’Odontostomatologie, Université des Sciences, des Techniques et des Technologies, Bamako, Mali

**Keywords:** Motivation of health workers, Performance-based financing, District hospital, expectations, Frustration, Mali

## Abstract

**Background:**

Performance-Based Financing (PBF), an innovative health financing initiative, was recently implemented in Mali. PBF aims to improve quality of care by motivating health workers. The purpose of this research was to identify and understand how health workers’ expectations related to their experiences of the first cycle of payment of PBF subsidies, and how this experience affected their motivation and sentiments towards the intervention. We pose the research question, “how does the process of PBF subsidies impact the motivation of health workers in Mali?”

**Methods:**

We adopted a qualitative approach using multiple case studies. We chose three district hospitals (DH 1, 2 and 3) in three health districts (district 1, 2 and 3) among the ten in the Koulikoro region. Our cases correspond to the three DHs. We followed the principle of data source triangulation; we used 53 semi-directive interviews conducted with health workers (to follow the principle of saturuation), field notes, and documents relating to the distribution grids of subsidies for each DH. We analyzed data in a mixed deductive and inductive manner.

**Results:**

The results show that the PBF subsidies led to health workers feeling more motivated to perform their tasks overall. Beyond financial motivation, this was primarily due to PBF allowing them to work more efficiently. However, respondents perceived a discrepancy between the efforts made and the subsidies received. The fact that their expectations were not met led to a sense of frustration and disappointment. Similarly, the way in which the subsidies were distributed and the lack of transparency in the distribution process led to feelings of unfairness among the vast majority of respondents. The results show that frustrations can build up in the early days of the intervention.

**Conclusion:**

The PBF implementation in Mali left health workers frustrated. The short overall implementation period did not allow actors to adjust their initial expectations and motivational responses, neither positive nor negative. This underlines how short-term interventions might not just lack impact, but instil negative sentiments likely to carry on into the future.

## Background

The performance of health workers is a determining factor in the provision of health care. It influences the quality of the health services offered and the level of health of the population [1, 2]. Several factors influence the performance of health workers in low- and middle-income countries (LMICs): financial and non-financial incentives, career development, continuing education, the state of infrastructure, the availability of human and material resources, the management and leadership skills of managers, and job satisfaction [3].

Despite the fact that motivation is one of the key factors in job performance, it remains a challenge in LMICs. There are several theories about motivation at work [4, 5]. Ryan and Deci define motivation as the drive or energy to act towards an objective [6]. In Mali, the Ministry of Health postulates that health workers working in the public system are very poorly motivated, regardless of whether they work in rural or urban areas [7]. Reasons for their lack of motivation are diverse: insufficient remuneration, poor physical working conditions, lack of professional training, lack of consideration, and poor organization and distribution of work [7, 8].

In LMICs, several pilot projects of performance-based financing (PBF) have been implemented with the aim of improving health service provision. One assumed result is that by linking financial rewards to predefined performance objectives, health workers will be more motivated to perform well [9]. Over the past several years, the architecture of health financing in LMICs has evolved significantly in Africa [10, 11], with PBF being one of the most important, but not the only new introduction to the health financing landscape. While PBF aims to strengthen the capacity of health systems in a sustainable manner, this is not always the case [12, 13]. Many initiatives financed by international donors are short-term interventions with the desire to have a rapid impact to the detriment of medium- and long-term interventions [10, 14, 15].

A number of studies have looked into the effects of PBF on health worker motivation in Africa [12, 16–20]. However, there is not yet scientific consensus on these effects, their direction and strength, their exact mechanisms, nor on developments over time [20–25]. In regards to the latter, the majority of existing studies were carried out fairly long after the start of implementation (12 months in Burkina Faso, [17], 18 months in Nigeria [26], 24 months in Malawi [20]). Studies suggest that the initial months of implementation were particularly crucial in shaping expectations and sentiments towards PBF that would carry through the entire implementation period. The long recall periods over an extensive implementation period likely distorted memories particularly in relation to the early implementation days.

To our knowledge, in Africa, the gap between what health workers expect from the PBF and the reality is very little studied, especially in relation to the early days of implementation. In Mali, PBF was only implemented for 8 months in total. The purpose of this research was to identify and understand how health workers’ expectations related to their experiences of the first cycle of payment of PBF subsidies, and how this experience affected their motivation and sentiments towards the intervention. We pose the research question, “how does the process of PBF subsidies impact the motivation of health workers in Mali?”

## Methods

### Study setting

Mali is a country in West Africa with an estimated population of 19,658,031 inhabitants as of 2019 [27]. It is subdivided into eight regions and the district of Bamako. In 2012, the country experienced a political, security, and institutional crisis following the occupation of the northern regions by armed groups, and the coup d’état of March 22 [28].

Between June 2016 and February 2017, a PBF pilot project took place in ten health districts in Mali’s Koulikoro region, as one of the sub-components of the Ministry of Health’s Strengthening Reproductive Health Project, funded by the World Bank. This project supports the strengthening of the health system through three components: (i) strengthening the supply and quality of reproductive health services; (ii) increasing the demand for reproductive health services; and (iii) strengthening social responsibility, project management, and monitoring-evaluation.

The objective of the pilot PBF project nested within this overall project was to increase the use of quality reproductive health services by increasing the motivation, responsibility, and accountability of service providers to achieve results [29]. For the implementation of the project, the Ministry of Health entered into a service contract with a specialized PBF agency, referred to as the Consortium (Royal Tropical Institute, Clinique de Gestion et d’Innovation des Connaissances, and Cordaid). Initially, the project was planned for 2 years [30]. For politico-administrative reasons, however, it ultimately only ran for 8 months. Several activities were carried out during its short implementation: training of actors, development of results plans, signing of contracts, production of results by health centers, reporting and verification of results in terms of quantity and quality of care, community verification, measurement of user satisfaction, and payment for results of one three-month cycle [31].

Mali’s health system is pyramidal at three levels (peripheral, intermediate, and central). At the central level, there are national hospitals and university hospitals. At the intermediate level are reference health centers (Centre de santé de reference [CSRéf]) or district hospitals (DH). At peripheral level, community health centres (Centres de santé communautaires [CSCom]) provide primary-level care. This PBF pilot project concerned only DHs and community health centres in the Koulikoro region. The DHs and community health centres were to be paid on the basis of results concerning reproductive health indicators (family planning, delivery by qualified personnel, pre- and post-natal care) [29]. Verification of results in terms of quantity and quality to authorize the payment of subsidies (which is the term locally used for the PBF payment, synonymous with incentives or bonuses) were supposed to be carried out on a monthly basis. However, this schedule was not adhered to in practice, with verification activities delayed by 7 to 6 months depending on the hospital. Local NGOs carried out community verification (CV) in the last month of implementation (February 2017), and factored it into the one payment made into the facilities’ bank accounts in February 2017.

In the DHs, at least 40% of the total amount of subsidies was reserved for investments and purchase of equipment, and a maximum of 60% could be used as individual subsidies for health workers. In community health centres, at least 60% of the total subsidies had to be invested or used for purchasing equipment, and 40% could be used as individual subsidies for health workers.

In regard to the individual subsidies, a fixed portion of 28% was to be distributed according to the socio-professional category of the health workers, and a variable portion of 72% according to individual performance over a three-month period [32]. The calculation of the performance percentage was made on the basis of the tasks recorded in each individual’s engagement form [32]. The fixed portion was calculated on the basis of each health workers’ salary category. For health workers whose wages were lower than the guaranteed minimum interprofessional wage, the amount was calculated by dividing the net monthly wage by an index value of 335 CFA francs (US $0.56) [32]. PBF provided an excel spreadsheet to facilitate the calculation of the total amounts to be received by each facility staff.

Important discrepancies existed between what was foreseen at the outset of implementation and stipulated in the procedures manual, and what was actually implemented. Most importantly, while two cycles of service purchasing (including verification and payment) were planned, only one cycle (3 months) was completed during the 8 months of implementation.

### Study design

We adopted a qualitative approach, based on an estimate of multiple case studies [33]. Our study is part of a larger research program in the context of which the cases were selected. For our study, we chose three DHs (DH 1, 2 and 3) in three health districts (district 1, 2 and 3) among the ten in the Koulikoro region. Our cases correspond to the three DHs. District 1 was more of a rural region, and had participated in the PBF pre-pilot project from February 2012 to December 2013, unlike Districts 2 and 3. District 2 was chosen because it was more urban than the other two districts. District 3 was selected because it was intended to link PBF with the community-based health insurance already existing there. These numbers of cases take into account our resource constraints, but provide sufficient representation of the diversity of contextual situations conducive to the analytical generalization specific to the case studies [34].

### Sampling of participants

Participants were purposively selected to reflect all socio-professional categories and sub-profiles working at the DH, and thereby achieve maximum variation in opinions and experiences of PBF. Once in the field, TZ selected the respondents in a purposive manner to represent all stakeholder groups (Table 1) and in consideration of their availability at the time of the survey. Once he had reached saturation at a study site, TZ proceeded with data collection at the next district hospital. Of the resulting sample of 53 respondents, 34 were male and 19 were female.

**Table 1:** Respondents in the study

Respondents		DH1	DH2	DH3	Total
Socio-professional category	Profiles				
A	Specialist doctor	1	1	0	2
	General physician	0	2	2	4
	Pharmacist	1	0	0	1
	Medical Assistant	3	1	1	5
B	Senior Health Technician	5	1	4	10
	Health technician	4	3	0	7
	State nurse	2	1	3	6
	Midwife	2	1	2	5
	Financial Controller	0	1	1	2
C	Obstetrician Nurse	2	1	0	3
	Executive Secretary	0	1	0	1
D	Ambulance driver	0	2	2	4
E	Hygienist	0	1	0	1
	Security guard	0	0	2	2
**Total**		20	16	17	53

### Analysis framework and data collection

We used Paul and Robinson’s classification of motivation types [35]. According to this classification, health worker motivation can be broadly categorized into three types: financial, social, and internal (Table 2).

**Table 2:** Paul and Robinson’s classification of health worker motivation types

Types of Motivations	Description
Financial motivation	Performance driven by desire to obtain subsidies/bonuses, salary, or other financial benefits.
Social motivation	Performance driven by social relationships in the workplace that promote feeling of approval and/or acceptance of others, which are related to the need to identify with a group, to adhere to standards. The concept of a sense of justice in the sense of fair treatment is central.
Internal motivation	Performance is driven by desire to act in accordance with one’s values and beliefs, independently of personal benefits, in order to feel that one’s behaviour has meaning and/or to enjoy doing one’s job on a daily basis, the various tasks related to that job, having an interest in those tasks in itself.

The PBF pilot project ended in February 2017. According to DHs, the payment of the first and only round of PBF subsidies was made approximately 5 to 6 months (July to August 2017) after the end of the project. From October 2, 2017 to November 20, 2017, TZ conducted 53 semi-structured interviews with health workers in three DHs in the Koulikoro region—about 2 months after the payment of subsidies. TZ, AC, AF, JL, and VR developed the data collection tools. We used two semi-structured interviews guides: one for the District Health Manager, and another for the rest of the staff. We followed the principle of triangulation advocated in the case studies by using multiple lines of evidence [33]. The different data sources used were: the 53 semi-directive interviews conducted with the different groups of actors, the TZ field notes, and documents relating to the distribution grids of subsidies for each DH. We used subsidy distribution grids to triangulate respondents’ information on the amount of subsidies obtained and the criteria taken into account when calculating subsidies in each DHs.

### Data processing and analysis

Research assistants transcribed the data verbatim, with quality monitored by the research team. TZ performed data coding using a code tree constructed by TZ, AC, AF, JL, and VR along Paul and Robinson’s classification of motivation types [35]. We followed a mixed deductive-inductive approach to content analysis based primarily on the predefined code tree, but allowed a few additional codes to emerge from the data as we went through the material. We used QDA Miner Lite for data coding. All authors discussed the resulting coding, and agreed on the emerging interpretation.

## Results

This section presents the main findings on the modalities and process of distribution of the first round of PBF subsidy payments, and its effects on the motivation of health workers.

### Motivation of health workers before the arrival of the PBF

The vast majority of respondents said they chose their profession out of vocation or love for their job, and that they loved working in their job before the arrival of PBF. In their opinion, being a health care worker is a noble profession in society. For health workers, contact with patients on a daily basis and the care they provide is a great source of inner satisfaction (internal motivation). However, many respondents cited difficulties in their working conditions (low salary, lack of equipment, retraining, and human resources) as hindering their ability to provide quality care to the population. At times, these difficulties negatively affected their motivation.

### Positive PBF effects on health worker motivation

Prior to the implementation of the PBF, the vast majority of health workers did not receive individual subsidies or other bonus payments. Respondents recognized that there was a change in their attitudes when their work started being valued by a financial subsidy (financial motivation). The majority of respondents equated receiving subsidies with both encouragement and incentive to do their job better. In addition, they perceived individual and hospital subsidies as a means to improving their difficult working conditions:

*“When there are (individual) subsidies, it changes people’s behaviour. In spite of the willingness, i.e. the willingness to work well, when there is money it always supports that. No matter what people say, money is a source of motivation. Today in a structure like the district hospital, it is the subsidies that can motivate the staff to do well. I don’t see anything else” [Specialist doctor, HD 2]*

It was not only the financial aspect of the PBF that affected their motivation, but PBF also increased internal motivation:

*“When you do a good job, you feel in yourself that you’ve done a good job, there’s satisfaction, that’s not material, but it makes you feel comfortable inside” [Senior Health Technician, HD1]*

According to some respondents, improved recognition of their work by their superiors was another factor contributing to their job satisfaction (internal motivation). Moreover, according to some respondents, PBF made it possible to better describe and distribute work tasks between health workers, contributing to better planning and organization of daily work.

### Development process of health workers’ expectations regarding subsidies

The vast majority of health workers were happy when the subsidies were announced with the introduction of PBF. However, after receiving their first PBF subsidies, respondents felt disappointed because they perceived a huge gap between the amounts hoped for and the amounts received. Most respondents found the amount of individual subsidy received was too low, and therefore not motivating. In addition, they perceived a discrepancy between the efforts made and the amount obtained (financial motivation). Disappointment with the subsidy amount varied from one socio-professional category to another. Specifically, disappointment was greater among health workers in the lower-level categories C, D, and E (hygienist, ambulance driver, security guard, etc.), because they placed more hope in the amount of the subsidies than those in higher-level categories A and B (doctors, pharmacists, and administrative staff). For example, in the three HDs, health workers in socio-professional categories A and B obtained between 20,000 CFA francs (US $32.99) and 30,000 CFA francs (US $49.49) as an individual subsidy for the one paid cycle, which corresponds to 3 months of the PBF. Those in categories C, D, and E had individual subsidy ranging from 3500 CFA francs (US $5.77) to 15,000 CFA francs (US $24.74):

*“I know of [a] Hygienist who went into debt because they said there’s PBF subsidies ... they got 3,500 CFA francs (US $5.85) as a subsidies... (Laughing) they were really discouraged.” [male ambulance driver, HD2]*

*“I felt a little discouraged about what I was given as payment. I told myself that I actually worked for three months and at the end of my effort I only received 24,000 CFA francs (US $40.14). For this reason, I planned to eventually do another activity, and leave the PBF for the benefit of this activity. For example, if I had a mission within the district, I will forcibly leave the follow-up here, because I know that in two or three days I will have 40,000 CFA francs (US $66.90). Because everyone is chasing after profit. Here I don’t think if someone has exceeded 30,000 CFA francs (US $50.17) or 40,000 CFA francs (US $66.90)” [Senior Health Technician, HD1].*

In addition, DH 1 health workers who had previously participated in the PBF pre-pilot project were more disappointed with the amount of individual subsidy received compared to DH 2 and 3, as the majority of them hoped for an amount on average equal to those they obtained during the PBF pre-pilot project, where higher subsidies had been paid.

### Effects of PBF implementation difficulties on the motivation of health workers

According to DHs, there was a 6 to 7 months delay in the distribution of subsidies. There are two reasons for this delay: administrative delays due to the complexity of the PBF process, and errors in bank processing. Respondents stated that they had not received any formal information on the reasons for the delay. The lack of transparency caused the circulation of rumors accusing those responsible of having embezzled subsidies. Some health workers felt that if subsidies are delayed, officials need to communicate the reasons better. Health workers’ suspicions of their managers reveal a lack of confidence in the management of finances.

In addition to creating a lack of confidence in the PBF mechanism among health workers, the delay in the disbursement of subsidies and its distribution also led to a lack of motivation among the workers. Health workers were informed individually by the hospital accounting department when the subsidies arrived. However, there was no meeting to inform the health workers about the overall amount of subsidies, individual subsidies, and hospital investment subsidies. A significant number of respondents stated that the subsidy sharing process was not transparent, which decreased motivation (social motivation):

“When they call you just to give you a sum of money, I can even say that the district hospital got billions and they only gave me 20,000 CFA francs (US $33.95) when that's not it, you have to be transparent because that's the best way” [Medical Assistant, HD1]*.*

Prior to the start of the project, the PBF agency established a key for the distribution of subsidies with several criteria to be used for the calculation of subsidies. Apart from the members of the district health management team (district health manager, manager, and unit heads), most health workers had only partial knowledge of these criteria. Respondents stated that details of the distribution of subsidies were not discussed in depth during the training sessions. The managers of DH 1 had a better grasp of how subsidies were distributed compared to those of DH 2 and 3 since they had already participated in the PBF pre-pilot project. In two DHs (DH 2 and 3), most of the distribution criteria were not applied when the subsidies were distributed. For example, in DH 2, some volunteer trainees and members of the management committee received unforeseen subsidies. Similarly, in DH 2 and 3, individual performance was not evaluated. This omission was possibly because the evaluation of individual performance had not been carried out. According to some hospital officials, since the PBF project had already ended, there was no longer a point in evaluating the health workers, and doing so would only create tensions within the teams. In contrast to this judgement, DH 1 health workers, where individual performance evaluation had taken place, stressed the importance of knowing their individual performance rating as an important element for morale:

“If I don't even know the grade I've got, I can really get an idea that somewhere they've taken something off, ... I can get fuzzy ideas.” [Senior Health Technician, DH 1]

As a result of the above issues, the vast majority of health workers felt that the subsidies were not well allocated (social motivation). In addition, they felt that the criteria considered when calculating the subsidies did not reflect everyone’s effort. In the absence of individual performance evaluations in DHs 2 and 3, there was a discrepancy in views among the different actors with regard to the criteria that should have been considered for the sharing of subsidies. Ideas varied in relation to the socio-professional category of health workers. In particular, for the majority of category C, D, and E health workers, subsidies should have been shared equally among all health workers without distinction of socio-professional category. However, the majority of category A and B health workers were against this egality principle. In their opinion, only the socio-professional category should have been considered, not individual performance. This difference of views among health workers sometimes led to tensions. Some health workers of socio-professional categories C, D, and E reported that they work more than those of higher categories A and B, and therefore should have received the same or higher amounts. For example, in DH 2, a health worker refused to receive his subsidies because he considered the amount inadequate in relation to the effort made. Nevertheless, the vast majority of health workers did not openly express their dissatisfaction:

“the way we did it (the distribution of subsidies), even if they don't tell us we know there's frustration” [General physician, DH2]

## Discussion

Our study is the first in Mali to explore the expectations of health workers with regard to the first cycle of PBF subsidies payment, and its effects on their motivation. It is one of the few publications in French-speaking West Africa on the subject, and the first to explore dynamics of the initial days of PBF.

### PBF motivates but can also demotivate health workers

Our findings show that PBF can motivate health workers to increase effort at work. However, our findings show that if it is poorly adapted to the implementation context or poorly implemented, it can become a source of demotivation and thus produce the opposite effect to that initially intended. Our results show that the principle of the PBF mechanism is well received by health workers. For them, the fact that they benefitted from subsidies under PBF motivated them to improve their performance. However, difficulties in implementation had a negative impact on their motivation. The delay of PBF subsidies led to a lack of confidence among health workers both in the PBF mechanism and in their managers. Stakeholder confidence in the health system is an important factor and is indicative of interpersonal relationships within the organization [36, 37]. In South Africa, Gilson et al. showed that health providers’ trust in their manager and the employing organization are important factors influencing performance [38]. Within the framework of the implementation of the PBF in Mali, preserving trust of the providers towards the PBF and those in charge would have required more effective communication and transparency in the organizational and financial management of the hospitals.

Two of the DHs had not applied staff performance evaluation as a criterion for calculating individual subsidies. In Mali, the modalities for sharing PBF subsidies varied by setting and among DHs. Several studies (in Benin [12], Tanzania [18], and Burkina Faso [17, 39]) show that the methods of distribution of PBF subsidies in health centers are heterogeneous. Interestingly, in these studies suggesting that whatever the modalities chosen, there was sometimes tension among providers within the health centers. In Tanzania, for example, the methods for distributing PBF incentives varied among health units in health centers (60% to be shared among reproductive health staff, 30% among other staff, and 10% at the health centers) [18], which led to tension. In Mali, since the PBF intervention is performance-based, the failure to choose this performance criterion for the calculation of subsidies was perceived as unfair by health workers. Burkina Faso [17, 39], Benin [12], and Tanzania [18] all experienced similar feelings of inequity with regard to the distribution process and the criteria chosen for calculating individual PBF subsidies. In Tanzania, perceived unfairness by health workers negatively affected work motivation and undermined teamwork between services [18]. In Burkina Faso, in some health centres, managers disregarded the planned performance criteria, which was unfair to health workers [17, 39].

In Mali, the lack of feedback for health workers on their performance evaluation under PBF meant that the program missed out on the opportunity to improve internal motivation, i.e., recognition of the tasks they perform in their work. In Burkina Faso, health workers appreciated the feedback during the implementation of the PBF because it enabled them to improve recognition of their work, and they made an effort to change following negative feedback [17]. However, in Tanzania, health workers reported insufficient feedback on their performance evaluation [40]. This lack of feedback resonates with the psychological literature on motivation, which stresses that the implementation of an effective performance evaluation system is essential to promoting internal motivation at work [41]. In particular, the literature shows that perceived fairness in the performance appraisal process can affect workers’ relationship with managers, their performance, their job satisfaction, and their well-being [42–44].

The delay of PBF subsidies within hospitals led to rumors circulating among some health workers, accusing officials of embezzling the subsidies. This circulation of rumors about PBF subsidies shows the importance that health workers attach to subsidies issues, specifically to their timely payment to avoid unfavorable incidents [45] or if persisting, obstacles to the successful delivery of health interventions [46].

Recourse to a logic of financial profitability is increasingly used to finance public health policies [47]. By using performance criteria, the PBF mechanism illustrates a contractual approach based on a logic of financial profitability. PBF is a form of financialization of public health policies;in other words, *“the penetration of financialized logics and forms of evaluation in the formulation and implementation of policies, even when these do not concern the financial sector”* [48]. In addition, the financialisation of public policies leads to the creation of new financial circuits [48]. The implementation of the PBF has brought additional money into hospitals, on top of the money usually invested. The implementation of PBF brings a new flow of money into the health care system. This new financial circuit completely changes the financing architecture that existed, where funds were injected by donors into the health system via the central level through different levels before landing in the health centres at the local level. This new financial circuit brought about by the PBF changed and structured the relationship between the beneficiaries (hospitals and health workers) and the health financing circuit. This new financial circuit allows for a market relationship (signing contracts, exchanging money, and setting the conditions for distributing the money) between a buyer (PBF’s specialized agency in our case) and suppliers (hospitals and health workers). The process of distributing PBF subsidies to hospitals in Mali shows us how PBF as a new health financing mechanism has changed the financing circuit of hospitals.

### Discrepancy between health workers’ expectations of PBF subsidies and the reality of implementation

A systematic review showed that the implementation of health initiatives requiring change in the health systems of LMICs is often complex and difficult [49]. There is often a gap between what was planned and what was achieved [50]. Moreover, public health policies are sometimes more incoherent and ineffective in the context of Sub-Saharan African [51]. Our study showed that the expectations of healthcare professionals regarding the individual subsidies was not met. When health workers saw how things worked in reality, it led to frustration and disappointment. It is important to differentiate relative frustration, as in our study (ie frustration in relation to expectations) from absolute frustration. Corcuff defines relative frustration as: *“a state of tension between expected and denied satisfaction, resulting in dissatisfaction, feeding a potential for dissatisfaction and collective action”* [27]. The frustration of the health workers in our study, therefore, stems from their comparison of what they have achieved in terms of earnings and socially constructed expectations. We believe that in Mali, health workers’ expectations of the PBF were too high during the pilot project; the majority of the workers expected at least the same amount that they obtained during the pre-pilot PBF project. They did not account for the fact that the PBF pilot project had fewer paid indicators compared to the pre-pilot project, and that the indicator purchasing scales were higher during the pre-pilot project compared to the pilot project. It is unclear whether their expectations were unreasonable, or grounded in lack of information about differences between the two schemes. Interestingly, in Tanzania, health workers were reporting positive – but in retrospect slightly unrealistic – expectations of the intervention even without “precedent” as in Mali. They felt that the possibility of future financial incentives should strengthen their motivation [42].

With respect to motivational and behavioural effects, we observed enthusiasm at the beginning of the implementation of the PBF intervention. We believe that the motivational and behavioural effects of the early days may have an impact on the long-term motivation of the actors. Motivation is a dynamic process; it is important to understand the changes in this dynamic process and their causes. Actors adjust their motivational expectations and reactions, both positive and negative, over time. Even if some problems persist when implementing complex initiatives such as PBF, positive and/or negative motivational reactions normalize or diminish over time. Unfortunately, in Mali, this normalization was not possible given the short duration of the project. Seppey and al. further demonstrated the lack of sustainability of the PBF in Mali [13]. The same observation was made by Antony and al. who, in their study in Benin, noted the lack of sustainability and institutional anchoring of the PBF pilot projects [12]. Several studies show that the vast majority of development projects initiated by NGOs and donors in African countries are unsustainable or low [13, 51]. However, short-term interventions are increasingly prioritized by donors at the expense of medium- and long-term interventions in LMICs [10]. In Mali, PBF certainly was one of those interventions that ended quickly without being able to rectify some of the negative effects due to its initial implementation challenges. When implementing development projects in the context of LMICs, it is necessary to analyze the frustrations that arise in the first days of interventions in order to readjust the projects. It is essential to ensure the sustainability of these projects from the design stage to facilitate the overall strengthening of health systems. Based on our experience, we propose that policies should have three priorities: (i) they should prioritize long-term projects where implementing actors will have more time to identify and correct implementation errors, and better adapt them to local contexts; (ii) they should aim to reduce the negative effects (delay in the payment of subsidies, lack of transparency in the subsidies sharing process, and the feeling of inadequacy between the work provided and the amount of subsidies obtained) that can demotivate health workers; and (iii), in baseline studies of a project aimed at motivating health workers, they should clearly identify workers’ expectations with regard to subsidies so as to better adapt the project and the communication of the project.

### Limitation of the study

Our research has certain limitations. First, the experience in hospitals during the implementation of the PBF and the opinions of the sampled health workers are not necessarily representative of all ten health districts in Koulikoro Region. Second, the data collection was carried out in a cross-sectional and retrospective manner. Some of the frustrations observed during data collection may have overshadowed the initial positive experiences with the intervention. Third, the subsidies were distributed several months after the PBF pilot project was terminated. As a result, some of the deviations observed during the subsidies sharing process may have been different if the implementation continued. Fourth, PBF was a donor-funded development project, even if the study was conducted independently. Thus, the interviews may have included a social desirability bias or prompted respondents to have a normative discourse. Response bias could include strategic responses from respondents who thought that their answers might influence the possible return of PBF. To reduce response bias, we have insisted on our independence from the PBF implementing agency in Mali.

## Conclusion

Our study showed that the PBF pilot project had both positive and negative effects on health worker motivation. Because there was not enough time for the actors to adjust their initial motivational expectations and reactions, PBF ended in frustration for many health workers built up during the first days of the intervention. Therefore, the motivational and behavioural effects of the first few days can have an impact on the long-term motivation of the actors.

## Data Availability

The data sets used and analyzed in this study are available from the corresponding author upon reasonable request.

## Abbreviations

CV: Community verification
DHs: District hospitals
LMICs: Low- and middle-income countries
PBF: Performance-based financing

## References

[1] OMS, éditeur. Travailler ensemble pour la santé. Genève: Organisation Mondiale de la Santé; 2006. 213. (Rapport sur la santé dans la monde).

[2] OMS (2010). Accroitre l’acces aux personnels de sante dans les zones rurales ou reculees grâce à une meilleure fidélisation recommandations pour une politique mondiale.

[3] Willis-Shattuck M, Bidwell P, Thomas S, Wyness L, Blaauw D, Ditlopo P (2008). Motivation and retention of health workers in developing countries: a systematic review. BMC Health Serv Res.

[4] Roussel P (2000). La motivation au travail - concept et théories.

[5] Maugeri S (2013). Théories de la motivation au travail.

[6] Ryan RM, Deci EL (2000). Intrinsic and Extrinsic Motivations: Classic Definitions and New Directions. Contemp Educ Psychol.

[7] Ministère de la sante du Mali (2009). Plan de développement des ressources humaines 2009-2015.

[8] Van Dormael M, Dugas S, Kone Y, Coulibaly S, Sy M, Marchal B (2008). Appropriate training and retention of community doctors in rural areas: a case study from Mali. Hum Resour Health.

[9] Fritsche GB, Soeters R, Meessen B (2014). Performance-based financing toolkit.

[10] Richard F, Hercot D, Ouédraogo C, Delvaux T, Samaké S, van Olmen J (2011). Sub-Saharan Africa and the health MDGs: the need to move beyond the “quick impact” model. Reprod Health Matters.

[11] Mwisongo A, Nabyonga-Orem J (2016). Global health initiatives in Africa – governance, priorities, harmonisation and alignment. BMC Health Serv Res.

[12] Antony M, Bertone MP, Barthes O. Exploring implementation practices in results-based financing: the case of the verification in Benin. BMC Health Serv Res. 2017;17(1) [cité 26 févr 2018] Disponible sur: http://bmchealthservres.biomedcentral.com/articles/10.1186/s12913-017-2148-9. 10.1186/s12913-017-2148-9 PMC5348780 28288637

[13] Seppey M, Ridde V, Touré L, Coulibaly A. Donor-funded project’s sustainability assessment: a qualitative case study of a results-based financing pilot in Koulikoro region, Mali. Globalization and Health. 2017;13(1) [cité 1 oct 2018] Disponible sur: https://globalizationandhealth.biomedcentral.com/articles/10.1186/s12992-017-0307-8. 10.1186/s12992-017-0307-8 PMC5721604 29216877

[14] Nations Unies. Projets à effet rapide pour les communautés [Internet]. [cité 26 mai 2020]. Disponible sur: https://peacekeeping.un.org/fr/quick-impact-projects-communities.

[15] Minusma. Projets à impact rapide (QIPS) [Internet]. [cité 26 mai 2020]. Disponible sur: https://minusma.unmissions.org/projets-%C3%A0-impact-rapide-qips.

[16] Kane S, Gandidzanwa C, Mutasa R. Coming full circle: how health worker motivation and performance in results-based financing arrangements hinges on strong and adaptive health systems. Int J Health Policy Manag. 2019;8(2):101-11. 10.15171/ijhpm.2018.98 PMC6462202 30980623

[17] Ridde V, Yaogo M, Zongo S, Somé P-A, Turcotte-Tremblay A-M. Twelve months of implementation of health care performance-based financing in Burkina Faso: A qualitative multiple case study. Int J Health Plann Manag. 2017; [cité 26 févr 2018]; Disponible sur: http://doi.wiley.com/10.1002/hpm.2439. 10.1002/hpm.2439 PMC5900741 28671285

[18] Chimhutu V, Songstad NG, Tjomsland M, Mrisho M, Moland KM. The inescapable question of fairness in Pay-for-performance bonus distribution: a qualitative study of health workers’ experiences in Tanzania. Glob Health. 2016;12(1) [cité 27 févr 2018] Disponible sur: http://globalizationandhealth.biomedcentral.com/articles/10.1186/s12992-016-0213-5. 10.1186/s12992-016-0213-5 PMC5123229 27884185

[19] Bertone MP, Lagarde M, Witter S. Performance-based financing in the context of the complex remuneration of health workers: findings from a mixed-method study in rural Sierra Leone. BMC Health Serv Res. 2016;16(1) [cité 26 févr 2018] Disponible sur: http://bmchealthservres.biomedcentral.com/articles/10.1186/s12913-016-1546-8. 10.1186/s12913-016-1546-8 PMC4952280 27435164

[20] Lohmann J, Wilhelm D, Kambala C, Brenner S, Muula AS, De Allegri M (2018). The money can be a motivator, to me a little, but mostly PBF just helps me to do better in my job.’ An exploration of the motivational mechanisms of performance-based financing for health workers in Malawi. Health Policy Plann.

[21] Aninanya GA, Howard N, Williams JE, Apam B, Prytherch H, Loukanova S (2016). Can performance-based incentives improve motivation of nurses and midwives in primary facilities in northern Ghana? A quasi-experimental study. Global Health Action.

[22] Fillol A, Lohmann J, Turcotte-Tremblay A-M, Somé P-A, Ridde V (2019). The Importance of Leadership and Organizational Capacity in Shaping Health Workers’ Motivational Reactions to Performance-Based Financing: A Multiple Case Study in Burkina Faso. Int J Health Policy Manag.

[23] Lohmann J, Souares A, Tiendrebéogo J, Houlfort N, Robyn PJ, Somda SM (2017). Measuring health workers’ motivation composition: validation of a scale based on self-determination theory in Burkina Faso. Hum Resour Health.

[24] Lohmann J, Houlfort N, De Allegri M (2016). Crowding out or no crowding out? A Self-Determination Theory approach to health worker motivation in performance-based financing. Soc Sci Med.

[25] Renmans D, Holvoet N, Criel B, Meessen B (2017). Performance-based financing: the same is different. Health Policy Planning.

[26] Ogundeji YK, Jackson C, Sheldon T, Olubajo O, Ihebuzor N (2016). Pay for performance in Nigeria: the influence of context and implementation on results. Health Policy Plann.

[27] World Bank. Population, total | Data - BANQUE MONDIALE : Données [Internet]. [cité 30 juill 2020]. Disponible sur: https://donnees.banquemondiale.org/indicator/SP.POP.TOTL?end=2019&start=2019&view=map.

[28] FMI. Cadre de stratégie pour la croissance et la réduction de la pauvreté du Mali. Washington, D.C: Fonds monétaire international; 2013. p. 386. Report No.: No. 13/111.

[29] Cissouma L, Sioro A, Koné O, Traoré K, Bulthuis S, Mommers P (2017). Rapport de Capitalisation FBR à la Malienne.

[30] World Bank. Mali - strengthening reproductive Health project. Washington, D.C.: World Bank Group; 2017. août [cité 7 mai 2019] p. 81. Report no.: ICR4095. Disponible sur: http://documents.worldbank.org/curated/en/542131502720462868/Mali-Strengthening-Reproductive-Health-Project.

[31] KIT, Clinique de gestion et d’innovation des connaissances, Cordaid (2017). RAPPORT FINAL DU PROJET PILOTE « PRSR/FBR » FBR-Financement Basé sur les Résultats – Approche pour accélérer les résultats en Santé de la Reproduction.

[32] Ministère de la Santé et de l’Hygiène Publique (2016). Manuel Opérationnel du Financement basé sur les résultats du projet PRSR-FBR.

[33] Yin RK. Case study research: design and methods. 4th ed. Los Angeles: Sage Publications; 2009. p. 219. Applied social research methods.

[34] Devaux-Spatarakis A, Gregot A (2012). Les défis de l’emploi de l’étude de cas en évaluation. caf.

[35] Paul E, Robinson M. Performance budgeting, motivation, and incentives. In: Robinson M, éditeur. Performance budgeting: linking funding and results. London: Palgrave Macmillan UK; 2007. 330-375. Disponible sur: doi: 10.1057/9781137001528_18.

[36] Ridde V (2005). Building trust or buying results?. Lancet.

[37] Gilson L (2005). Editorial: building trust and value in health systems in low- and middle-income countries. Soc Sci Med.

[38] Gilson L, Palmer N, Schneider H (2005). Trust and health worker performance: exploring a conceptual framework using south African evidence. Soc Sci Med.

[39] Lohmann J, Koulidiati J-L, Somda SM, De Allegri M. It Depends on What They Experience in Each Health Facility. Some Are Satisfied, Others Are Not. A MixedMethods Exploration of Health Workers’ Attitudes Towards Performance-Based Financing in Burkina Faso. Int J Health Policy Manag. 2020;1 [cité 30 juill 2020] Disponible sur: https://www.ijhpm.com/article_3803.html. 10.34172/ijhpm.2020.61 PMC9056201 32610757

[40] Songstad N, Lindkvist I, Moland K, Chimhutu V, Blystad A (2012). Assessing performance enhancing tools: experiences with the open performance review and appraisal system (OPRAS) and expectations towards payment for performance (P4P) in the public health sector in Tanzania. Glob Health.

[41] Gagné M, Forest J (2008). The study of compensation systems through the lens of self-determination theory: Reconciling 35 years of debate. Can Psychol/Psychologie Canadienne.

[42] Blader SL, Tyler TR (2005). How can theories of organizational justice explain the effects of fairness? In: Handbook of organizational justice.

[43] Sparr JL, Sonnentag S (2008). Fairness perceptions of supervisor feedback, LMX, and employee well-being at work. Eur J Work Organ Psychol.

[44] Grenier S, Chiocchio F, Beaulieu G (2012). Évaluation du rendement et motivation au travail : propositions de recherche pour une rétroaction sur le rendement qui favorise la satisfaction des besoins psychologiques fondamentaux. mi.

[45] Bonhomme J, Bondaz J (2017). L’offrande de la mort: une rumeur au Sénégal.

[46] Kaler A (2009). Health interventions and the persistence of rumour: the circulation of sterility stories in African public health campaigns. Soc Sci Med.

[47] Gabas J-J, Ribier V, Vernières M (2017). Présentation. Financement ou financiarisation du développement ? Une question en débat. Mondes en développement.

[48] Chiapello È (2017). La financiarisation des politiques publiques. Mondes en développement.

[49] Corcuff P (2009). Frustrations relatives. Dictionnaire des mouvements sociaux.

[50] Olivier de Sardan J-P, Ridde V, éditeurs. Les spécificités des politiques publiques et des systèmes de santé en Afrique sahélienne. In: Une politique publique de santé et ses contradictions: la gratuité des soins au Burkina Faso, au Mali et au Niger. Paris: Éditions Karthala; 2014. 15–30. (Hommes et sociétés).

[51] Mallé Samb O, Ridde V, Queuille L (2013). Quelle pérennité pour les interventions pilotes de gratuité des soins au Burkina Faso ?. Revue Tiers Monde.

